# Increasing community capacity to improve the implementation of Health Promoting Schools: barriers and facilitators from the FLASH intervention

**DOI:** 10.1093/heapro/daad115

**Published:** 2023-09-30

**Authors:** Bonnie Maria van Dongen, Monica Antonia Maria Ridder, Loïs Wolters, Ingrid Hendrika Margaretha Steenhuis, Carry Mira Renders

**Affiliations:** Department of Health Sciences, Faculty of Science, Vrije Universiteit Amsterdam, Amsterdam Public Health Research Institute, van der Boechorststraat 7, 1081 BT Amsterdam, The Netherlands; Human Movement and Education Division, Windesheim University of Applied Sciences, Campus 2, 8017 CA Zwolle, The Netherlands; Department of Health Sciences, Faculty of Science, Vrije Universiteit Amsterdam, Amsterdam Public Health Research Institute, van der Boechorststraat 7, 1081 BT Amsterdam, The Netherlands; Department of Health Sciences, Faculty of Science, Vrije Universiteit Amsterdam, Amsterdam Public Health Research Institute, van der Boechorststraat 7, 1081 BT Amsterdam, The Netherlands; Department of Health Sciences, Faculty of Science, Vrije Universiteit Amsterdam, Amsterdam Public Health Research Institute, van der Boechorststraat 7, 1081 BT Amsterdam, The Netherlands; Department of Healthy Society, Windesheim University of Applied Sciences, Campus 2, 8017 CA Zwolle, The Netherlands

**Keywords:** Health Promoting Schools, community capacity, implementation, adolescents, leadership

## Abstract

Building community capacity is important for the successful implementation of a Health Promoting School. To identify how capacity building can be encouraged in secondary schools, four schools engaged in the Fit Lifestyle at School and at Home (FLASH) intervention for 3 years. This study explores barriers and facilitators that school personnel, parents and pupils experienced in the capacity-building process. Thirty-one stakeholders were interviewed. Transcripts were analysed thematically based on the five actions of the intervention: (i) appoint a Healthy School coordinator and build a team, (ii) determine ambitions, (iii) design and (iv) implement the action plan and (v) evaluate and improve. The time and support allocated to coordinators helped them evolve their role from executors of health-promotion activities to coordinators, instigators and gatekeepers of the implementation process. Participatory tools helped identify shared values among stakeholders to determine context-specific ambitions and leverage points for interventions. Coordinators indicated that they lacked the skills and authority to engage pupils and parents and to reach the broader community. Coordinators struggled with translating promising ideas into action plans of coherent and mutually supportive activities and embedding them into policy. Strong leadership of Healthy School coordinators, who focus on the capacity-building process and foster collaborative relationships, is essential to build community capacity. In this process, more guidance is needed on how to involve the broader community in various phases. Furthermore, coordinators can benefit from professional development to align jointly designed activities into a comprehensive action plan embedded into Healthy School policies.

**Contribution to Health Promotion**

- To create and sustain a Health Promoting School, personnel, parent and pupils must work together and form a community.
- We propose five non-linear actions that schools can engage in: appoint a coordinator and build a team, determine ambitions, design, implement and evaluate.
- Professional development of coordinators is important to become strong leaders who can be adaptive and flexible while coordinating the implementation process.
- Schools benefit from local expertise on implementation challenges, such as engaging pupils and parents, developing health policies and evaluation.

## INTRODUCTION

When secondary schools implement activities to encourage a healthy lifestyle among adolescents, the WHO advocates the use of the integral Health Promoting School (HPS) framework (WHO, 1986). This framework stimulates changes in the whole school system, combining health education with health policies and a favorable (social and physical) environment (Turunen *et al*., 2017). Despite increasing evidence for the effectiveness of this framework, sustainable implementation of local HPS initiatives remains challenging. Research shows that more effective and sustainable HPS initiatives are considered to be complex, multidimensional and embedded in more than one domain of school life (Stewart-Brown, 2006). Moreover, these initiatives are often the result of dynamic, non-linear and unpredictable processes. This complexity needs to be embraced rather than avoided (Keshavarz *et al*., 2010; Rowling and Samdal, 2011).

Previous research on indicators for sustainable implementation mentions a variety of different concepts, such as bottom-up involvement, tailoring and empowerment (Scheirer, 2005; Durlak and DuPre, 2008). Central to this is the ability of stakeholders, such as school personnel, pupils and parents, to navigate this complex implementation process (Mohammadi, 2010; Rosas, 2017). A promising way to empower stakeholders to jointly embrace such a process and adapt to emerging opportunities is by building community capacity (Millar *et al*., 2013; Swinburn *et al*., 2014). This entails the development of knowledge, skills, ownership, leadership, structures and systems on an individual and organizational level to develop and sustain context-specific solutions to problems in a way that helps stakeholders control their own environment (Hoyle *et al*., 2008; Bergeron *et al*., 2017). Capacity building has long been recognized as an important indicator of program success because it focuses on the competence of a community to tackle complex and constantly evolving (health) issues now and in future (Hawe *et al*., 1997). Despite this, there is no clear approach available to translate this strategy into practice (Liberato *et al*., 2011).

**Fig. 1:**
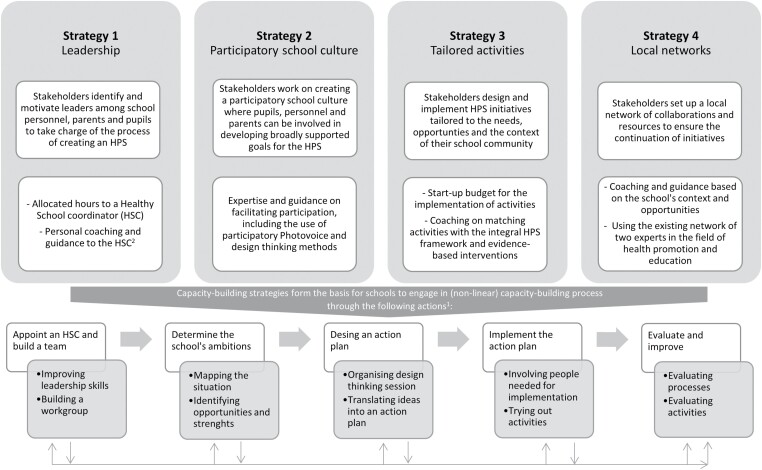
Overview of capacity-building strategies, inputs and process actions of the FLASH intervention. ^1^This figure illustrates the general design of the FLASH intervention, but the real-life implementation is not supposed to be linear. Because we followed a learning and iterative approach throughout this intervention and its evaluation, the process was carefully monitored and adjusted where contextual factors required. ^2^Two local experts in the field of health promotion and education, who already support schools in the Dutch Healthy School program as part of their regular job description, provided personal coaching. Both experts had knowledge of evidence-based interventions involving the Healthy School program and had connections to local organisations related to health promotion. Experts and the research team collaborated closely in the coaching of HSCs to establish an iterative process of co-creation to guide the HSC in the process of building community capacity.

In the context of the HPS framework, the importance of building community capacity is recognized in the starting principles considering that a Health Promoting School is described as a school that is *constantly strengthening their capacity as a setting for healthy living, learning and working* (WHO and UNESCO, 2021). Building community capacity therefore involves the ability to develop, implement and sustain an integral set of actions and activities that promote a healthy lifestyle. In the Netherlands, the HPS framework is translated into Healthy School program. This program differentiates four pillars (education, environment, policy and signaling), and is considered the standard if schools want to work on health and well-being. Although this program includes the availability of several promising health-topic-specific interventions of which sustainable implementation is encouraged, in practice this has proven difficult (Schaap *et al*., 2018). The Fit Lifestyle at School and at Home (FLASH) intervention was set up to investigate a different emphasis towards implementing the Dutch Healthy School program, namely a focus on building community capacity (van Dongen *et al*., 2019). Between 2016 and 2019, together with stakeholders from four secondary schools, we explored what building community capacity can entail when aiming for a sustainable implementation process, following a learning and adaptive approach to capture and accommodate changes in the system and to allow for feedback and emergent outcomes (Eoyang and Oakden, 2016). To guide the capacity-building process, four theory-based strategies were developed during FLASH: identifying and motivating leaders, promoting a participatory school culture, designing and implementing tailored health-promotion activities and creating a local network (Plested, 2006). Working on these strategies was considered a continuous process for stakeholders in each school, operating under the assumption that strategies can strengthen each other (Millar *et al*., 2013). To facilitate the capacity-building process, each school was provided with several inputs for each strategy (Figure 1).

A previous study evaluated the impact of FLASH on these capacity-building strategies (van Dongen *et al*., 2022); it revealed that the level of community capacity increased in all participating schools. Most notable improvements were seen in leadership. Schools also improved on participatory school culture and tailored activities. Moreover, this study described the adoption and implementation of each strategy (see Supplementary Appendix 1 for an overview). Experiences of stakeholders highlighted that building community capacity was considered beneficial to evoke a process of change, but requires time and effort as stakeholders must become acquainted with new roles and responsibilities. During this evaluation process, we observed five actions that schools engaged in to work on the four strategies: (i) appoint a Healthy School coordinator (HSC) and build a team, (ii) determine ambitions, (iii) design an action plan, (iv) implement an action plan, and (v) evaluate and improve. These actions were not self-contained, linear steps. Instead, actions were dynamic and non-linear in nature where opportunities in one action could lead to (expected and unexpected) opportunities for other actions in the capacity-building process. For example, an HSC could have started with building a team, but by together determining their ambitions new opportunities for important leadership roles could emerge. The current study aims to further identify barriers and facilitators that stakeholders experienced within these actions in order to explore how schools can invest in navigating the process of building community capacity.

## METHODS

### Study design

A qualitative study was performed to explore barriers and facilitators in the capacity-building process during the FLASH intervention. Semi-structured evaluation interviews were conducted between July 2019 and December 2019 with the following stakeholder groups: school personnel, parents and pupils. Ethical approval was provided by the Dutch Medical Research Involving Human Subjects Act (Medical Ethics Committee of Amsterdam UMC, VUmc location, reference number 2016.352).

### Setting

Four schools belonging to the same regional educational partnership in the northeastern region of the Netherlands participated in the FLASH intervention. Schools were recruited by this partnership, which was a collaborator in the FLASH intervention. As this partnership prioritized two health topics of the Healthy School program, namely ‘Nutrition’ and ‘Exercise and Sports’, FLASH was demarcated to these two topics. Schools were recruited based on their interest to also prioritize these two topics and their willingness to commit to the intervention and evaluation study. The school board had to be willing to facilitate (new) health promotion activities and to appoint a staff member to coordinate the FLASH intervention. The partnership was encouraged to recruit schools operating under varying contextual factors (size, location, educational tracks) to gain insight into how the process of building community capacity works in different contexts but on the same substantive health topics and with the same inputs to facilitate this process. Schools were eligible to participate if they offered pre-vocational education. In the Netherlands, pupils entering secondary education are streamed according to aptitude into three forms of schooling: pre-vocational (*vmbo*), professional (*havo*) and pre-university (*vwo*) (MECS, 2020). About half of them are tracked into the *vmbo* track. This is classified as a lower education level, which is identified as a risk factor for unhealthy behavior (Van Leest and Verschuren, 2005; Kraaykamp *et al*., 2018). Two schools offered education exclusively in the *vmbo* track, and two schools additionally offered other tracks. The schools differed in size regarding number of pupils and in location regarding a rural or urban environment (Table 1).

**Table 1:** Characteristics of intervention schools and respondents

	School 1	School 2	School 3	School 4
**School characteristics**				
Educational tracks^a^	Exclusively offers ‘profiles’ subtrack of the *vmbo track*	Comprehensive school offers only ‘learning pathway’ subtrack	Exclusively offers ‘profiles’ subtrack of the *vmbo track*	Comprehensive school. Offers all subtracks, but only the ‘learning pathway’ can be completed until final examination.
Environment	Urban area, city center	Urban area, city center	Rural area	Rural area
Size, N pupils: total (pre-vocational pupils total)	2016: 520 2017: 638 2018: 612	2016: 711 (221) 2017: 842 (245) 2018: 962 (286)	2016: 228 2017: 199 2018: 160	2016: 305 (219) 2017: 297 (213) 2018: 294 (204)
**Respondent characteristics**				
Healthy school coordinators	Dutch language teacher (M)	PE teacher (M)	PE Teacher (M)	German language teacher and student care coordinator (F)
Management	Team leader (M)	School director (F)	Team leader (M)	Location leader (M)
Teachers	Economics teacher (F) Biology teacher (F)	Biology teacher (M) Student care coordinator (F)	Care and well-being teacher (*N* = 2) (F, F) Biology teacher (F)	Biology teacher (F) PE teacher (M)
Supporting personnel	Communication/PR employee (F)	Canteen manager (F)	Receptionist/administrative assistant (F)	Custodian (M)
Parents	Member of parent council (F)	Parent of *vmbo* pupil (F)	—^c^	Member of parent council (M)
Pupils	Pupils grade 2 (*N* = 2) (1M 1F)	Pupil grade 2 (M) Pupils grade 4 (*N* = 2) (F, F)	—^c^	Pupils grade 2 (*N* = 3) (1M 2F)

^a^Pupils entering secondary education are streamed according to aptitude into one of three forms of schooling: pre-vocational (vmbo), professional (havo) and pre-university (vwo). After a two-year common basic curriculum, the vmbo track splits into subtracks (i.e., ‘learning pathway’ and ‘profile’), with selection according to ability, interests, and ambitions. Individual schools that provide more than one form of schooling (vmbo/havo/vwo), are classified as comprehensive schools.

^b^M = male, F = female.

^c^During the data collection period, contextual factors (such as changes in school locations and management, complex organization structures, drop in pupil population) created an unwilling atmosphere among pupils and parent to participate in requests not directly related to their own/child’s education. To prevent worsening of future relationships, the HSC decided to not make any further requests.

### Study population and recruitment

The study population consisted of representatives from each stakeholder group. We included stakeholders with various roles in the school community to ensure a complementary overview over the capacity-building process from different perspectives. Participants were identified in collaboration with the HSC. Purposive sampling was used to include stakeholders in the following roles in each school: (i) the HSC, who has an overview of all the processes regarding the Healthy School; (ii) a formal school leader; (iii) teaching staff; (iv) supporting staff (e.g. canteen personnel, custodian) involved with implementing activities during FLASH; (v) *vmbo-*pupils; and (vi) a parent or a guardian with a representative role in the school (e.g. a member of the parent council). All stakeholders were invited by the HSC and were sent an information letter by the researcher. Stakeholders provided written informed consent; additionally, written consent from a parent or guardian was obtained for pupils younger than 16.

### Data collection

The interview guide was based on the four capacity-building strategies central to the FLASH intervention. Central questions per strategy were as follows: ‘*How are leadership roles divided?*’, ‘*To what extent are various stakeholders willing to participate and how is this facilitated?*’, ‘*Which activities take place and how are they tailored to the school?*’ and *‘To what extent are local resources and collaborations set-up to continue efforts?*’. Participants were asked if they experienced any changes in the last 3 years in the school and what contributed or hindered potential changes. For interviews with pupils, questions were simplified and shortened. Supplementary Appendix 2 provides example questions for adult and pupil participants.

Adult participants were interviewed individually and face-to-face in an environment they chose: either in a quiet space at school or at their home. Interviews lasted between 45 and 100 min (average time 70 min). Pupils were interviewed in groups of two or three so that they could feel safe and at ease. Interviews took place during school hours and lasted between 35 and 60 min (average time 40 min). All interviews were audio recorded and transcribed verbatim. A member check was performed by sending the summary of the interview to the respondent.

### Data analysis

Transcripts were analyzed with MAXQDA 2020 software using a thematic content approach. Open coding was conducted to identify codes regarding stakeholders’ experiences about what facilitated and hindered the process of building community capacity. Two researchers first created codes independently and compared them afterwards (B.v.D. and L.W.). Based on the discussions, the codes were transformed into overarching interpretive themes. The two researchers met regularly to reach consensus and adjust themes while also maintaining reflective diaries throughout the coding process in order to evaluate subjective views. A third researcher (C.R.) helped reach consensus. Interpretive themes were then categorized into the observed actions in FLASH schools: (i) appoint an HSC and build a team, (ii) determine the school’s ambitions, (iii) design an action plan, (iv) implement the action plan and (v) evaluate and improve. Because actions were not self-contained or linear, themes could sometimes be categorized into several actions. We decided to categorize a theme in the action that was most influenced by the theme based on the context respondents provided throughout the interviews.

## RESULTS

Thirty-one stakeholders were interviewed across four schools with different characteristics (Table 1). The HSC in school 3 was not able to recruit pupils and a parent within the data collection period. Table 2 provides an overview of barriers and facilitators for each action in the FLASH intervention.

**Table 2:**
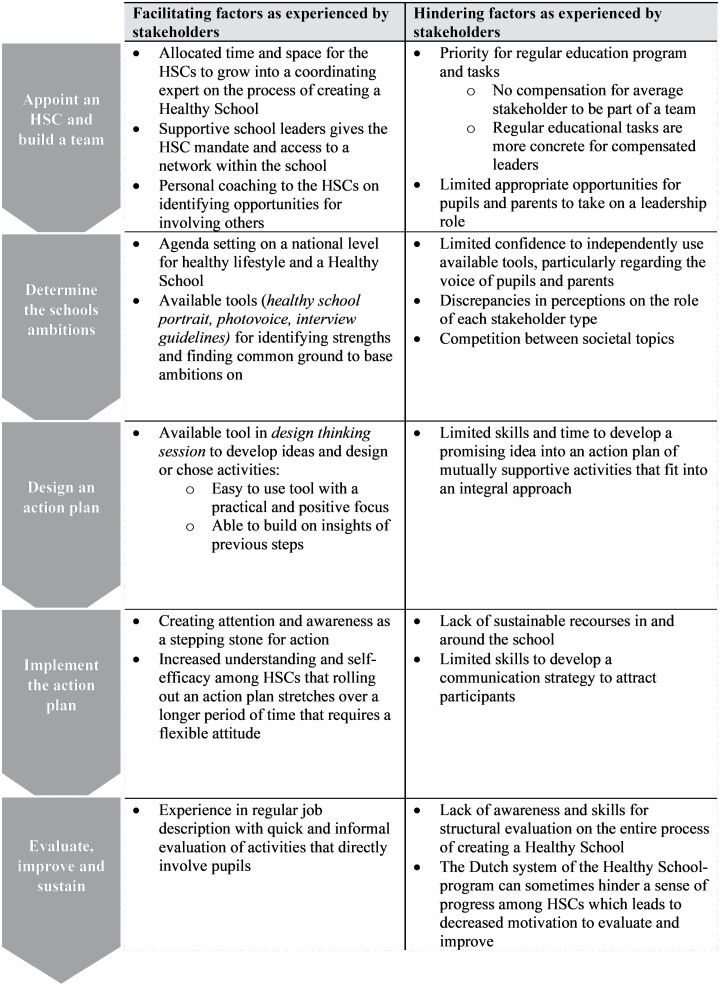
Overview of barriers and facilitators as experienced by stakeholders in the FLASH intervention

### Appoint an HSC and build a team

#### Facilitators

During FLASH, HSCs were allocated time and coaching that enabled them to grow in their role as coordinators of the HPS implementation process rather than remain executors of Healthy School activities. All HSCs increasingly considered implementation as a long-term process in which they constantly had to make efforts to identify stakeholders who could become involved in it.

Only during FLASH did I start to notice that things get moving because of the connections you make with people. It was not so much about the end goal but more about the process on how to get there and who I needed for that, that became important. (HSC, school 2)

Personal coaching triggered HSCs to broaden their search for opportunities to involve other stakeholders. All HSCs started by involving colleagues who were easy to approach because they already had connection to the field of health (e.g. biology or physical education teachers). With the support of an outside perspective, they also started to identify colleagues who had a specific role in the school that could be beneficial in the process of building community capacity (e.g. guidance counselors who take care of pupils’ welfare or PR employees who could communicate about Healthy School activities). HSCs felt that colleagues were more willing to participate when tasks were demarcated. Through the efforts of building a network of colleagues, HSCs became more visible in their schools and created flexible teams they could call upon when needed, as well as in other actions such as implementing action plans.

HSCs mentioned that having a supportive formal school leader facilitated the action of building a team, as they felt an increased sense of authorization to approach colleagues with their request to join the HPS team. Moreover, a supportive school leader could help in agenda setting, which also aided with other actions such as determining ambitions.

I now feel that it is my responsibility to constantly let people know that we are a Healthy School and that we act like that in everything we do. For example, during staff meetings I put the topic of the Healthy School on the agenda and I also start parent meetings by mentioning the Healthy School. (Location leader, school 4)

#### Barriers

According to multiple stakeholders in leadership positions, it remained difficult to involve colleagues since they lacked compensation (both time and financial) and had other priorities. This was the case even for colleagues who were intrinsically motivated to participate.

If I need someone for something structural, for example being part of a work group, I encounter resistance if they do not get compensated. (HSC, school 4)

Even if someone, such as the HSC, received compensation, it remained difficult to prioritize this responsibility since this role was not as clearly formulated than educational tasks.

HSCs mentioned they experienced even more difficulties involving parents and pupils. Even if a leading role was identified for pupils or parents in certain activities, HSCs felt that they did not have the authority to ask them to take it up. For example, they considered it beyond their responsibility to coordinate and communicate with a pupil or the parent council. These experiences also impacted other actions, as it became more challenging to involve a large number of parents and pupils in determining ambitions and developing action plans.

### Determine the school’s ambitions

#### Facilitators

Beneficial to determining the school’s ambitions was the development of the so-called Healthy School portrait, which described existing activities, pupils’ health behaviors, and strengths, opportunities and needs with regard to PA and healthy nutrition. Tools such as photovoice and interview guidelines helped stakeholders gain insight into each other’s perceptions in order to create such a portrait. Perceptions about the process of creating a Healthy School based on experiences with other health topics that stakeholders considered helpful in general were incorporated in these portraits. The portrait served as a communal starting point for discussing priorities, needs and ambitions that fit into the particular school’s context. For example, stakeholders in school 4 identified their organizational structure of offering elective modules as their school’s unique strength and found a shared value in the realization that this provided further opportunities to embed health into the curriculum. Stakeholders appreciated that they were asked for input regarding their school’s ambitions and developed an improved sense of ownership

If we had used the normal Healthy School-program, we probably would’ve felt that it would have been imposed from above and staff wouldn’t have supported it. But now we have strengthened what we had and made more of an effort together. (Custodian, school 4)

Moreover, multiple stakeholders felt that the national healthy lifestyle agenda supported a positive attitude among the school community when discussing ambitions for their Healthy School. This also created opportunities for other actions, such as motivating others to join the HPS team or more easily implement health-promotion activities.

#### Barriers

At the same time, stakeholders indicated that they were unsure of their own role and each other’s roles in the Healthy School community, including what degree of influence they had. This made them reluctant to participate actively in determining ambitions.

I think that teachers get more and more responsibilities these days. But you have to wonder: where is the line between responsibility for parents and for school? (Teacher, school 2)

Moreover, HSCs and teachers expressed little self-efficacy with regard to independently identifying shared values between stakeholders and opportunities in their school. Although FLASH tools, such as photovoice and the Healthy School portrait, were helpful, HSCs found it particularly difficult to bring out the voices of pupils and parents without coaching support. School staff also experienced that setting goals for a Healthy School competed with setting goals on other societal topics, such as climate change and citizenship. Although stakeholders were aware that these topics are sometimes intertwined and can strengthen each other, each topic often has its own agenda and a working group in a school. These barriers also influenced other actions, such as making agreements between leadership structures of different societal topics or conflicting views on the implementation of health-promotion activities.

### Design an action plan

#### Facilitators

Stakeholders appreciated the use of the design thinking tool to design activities. HSCs considered the tool easy to implement and a practical way to bring people together. By sharing and discussing various insights gained in determining their school’s ambition, stakeholders felt able to build upon their school’s strengths and unique context.

We developed a staircase challenge because we always hear from pupils that it is a prominent feature of this building. They are pretty steep and pupils always complain. But now we used it in a positive and fun way! (PR employee, school 3)

Stakeholders who participated in these sessions appreciated that it resulted in explicit, feasible ideas.

I think [the session] was a good way to discuss these things. It brought people together and created new ideas which were more feasible because people supported these ideas. (Parent, school 1)

#### Barriers

HSCs expressed difficulties translating promising ideas from design thinking sessions into an action plan of mutually supportive activities that fit into an integral approach. For example, stakeholders in school 1 were critical of the HSC’s proposal that focused on distributing water bottles but did not entail educational activities or structural changes in the canteen, which then influenced the commitment of the HPS team. Furthermore, HSCs indicated that they had trouble formulating actions aimed at influencing their school health policies. They were unsure of what a health policy specifically entailed besides rules and a general statement in policy documents that the school worked towards a Healthy School, and they questioned whether they had the mandate to develop policies. This also highlighted the need for them to actively invest in creating commitment from formal leadership (action ‘build a team). Additionally, they were not familiar with writing policy:

I know that we must say something about health policy in our school policy, but we have not written it down yet. As a school we also usually just do things, so I keep postponing it. (HSC, school 2)

### Implement the action plan

#### Facilitators

Stakeholders experienced that it was most beneficial to start with implementing activities that created positive attention and awareness because this could serve as a stepping stone for other actions and activities. For example, offering healthier sandwiches and snacks in the school canteen or distributing water bottles were visible activities with a direct link to healthier behavior. Generating enthusiasm for such activities was considered relatively easy because community members had a choice in whether to engage in healthier behavior. The HSC expected these activities to create a ripple effect, which would allow other actions from the action plan, such as introducing new health policies, to be implemented more easily. Moreover, HSCs noted that throughout other actions they developed in their role to become overarching coordinators in which they had gained a positive mindset and were more inclined to think in opportunities rather than barriers. Connections that they had made with colleagues in previous actions aided this mindset. Consequently, they adopted a flexible attitude and persevered in rolling out the action plan. For example, the HSCs in school 4 experienced delays in purchasing standing desks for pupils (due to a miscommunication with the school leader about responsibilities). While waiting for the desks, the HSC switched over, with the help of an enthusiastic colleague, to another activity that came up during the design thinking session.

My biology colleague was really assertive: she went to the [school] director and asked for a bit of money to buy vegetable seeds. With a few classes, she now started a greenhouse project, because we had one on school grounds that wasn’t used. This year is a pilot and next year she plans to use [municipal] funding to set-up a larger project for more pupils. (HSC, school 4)

#### Barriers

Stakeholders experienced barriers when changes affected the school’s available resources:

I would’ve liked to have rolled out the idea of setting up a classroom with bicycle chairs for pupils. But we just had a big increase in pupil numbers, which means I have no money left as I had to prioritise expanding our school to a different location. (School leader, school 2)

Stakeholders also indicated that they lacked the skills to set up a communication strategy to attract participants in activities, which affected outreach. For example, school 2 introduced a stamp card for buying healthy products in the school canteen, but pupils mentioned they were not aware that this stamp card existed. Consequently, the HSC reflected that other actions can still be improved upon, such as actively involving the PR employee in this school.

### Evaluate and improve

#### Facilitators

HSCs and teachers generally felt able to evaluate specific activities that directly involved pupils, which is part of their regular job. Curriculum-based activities in particular could be evaluated relatively quickly, easily and informally. When evaluating, they discussed how activities went and what could be done better the next time; this helped stakeholders visualize their goal of repeating activities on a yearly basis and improving each time, therefore also influencing the action of implementing the action plan.

We evaluate informally. For example with colleagues I discussed what worked and what didn’t. I write that down immediately and plan to do it differently next year. (HSC, school 3)

#### Barriers

Overall, stakeholders had little awareness and limited skills for structural reflection and evaluation. Specifically, actions relating to sustainable implementation of the Healthy School, such as health policies, ambitions or the position of the HSC in the organization, were rarely evaluated, which hindered the continuous loop of engaging in these five actions. When discussing evaluation, HSCs did mention the regular system of the Healthy School program. However, this system is aimed more at specific health promotion themes and did not necessarily provide HSCs with an insight into how the process of building community capacity could be improved. Moreover, working with this system seemed to demotivate HSCs for the task of creating a Healthy School community:

How it works now is that I have to tick off many items to show we’ve completed all the necessary requirements for a theme certificate. For nutrition, that takes a long time even though I know we’re doing a lot. Every time something is “wrong”, which is frustrating. (HSC, school 2)

## DISCUSSION

This study explored the barriers and facilitators that stakeholders experienced when building community capacity to create a Healthy School community. A prominent facilitator was the appointment of a designated HSC. With the assistance of personal coaching and supportive school leaders, HSCs were able to develop professionally as experts coordinating the process of creating a Healthy School. Thus, they focused more on involving other stakeholders in leadership positions than on organizing specific Healthy School activities on their own. Participatory tools, such as a photovoice and design thinking, helped stakeholders gain insight into the varying perspectives of community members and determine the strengths and shared values. This enabled stakeholders to jointly determine their school’s ambition and design an initial action plan. Visible activities that stimulated positive attention and created awareness among the school community were considered an important first step. According to stakeholders, these preliminary activities could potentially serve as a stepping stone for further action in the school. Involving pupils and parents remained challenging because expectations about the roles and responsibilities of different stakeholder groups were not clear to everyone. HSCs felt they lacked the skills and authority to involve the broader school community. Moreover, stakeholders in general had little awareness of and limited skills for certain tasks that support implementing the HPS framework as an integral approach (e.g. a set of comprehensive efforts aimed at education, environment and policy) (WHO, 2009). The capacity-building process particularly seemed to stagnate when promising ideas needed to be translated into an action plan of coherent and mutually supportive activities, including formulating a communication strategy and setting up a plan and resources needed to evaluate and improve the process of building community capacity.

Similar to other studies, our results highlight that having a strong leader is an important requirement in the process of building community capacity (Whelan *et al*., 2018) and consequently, in the sustainable implementation of the HPS (Herlitz *et al*., 2020). Many studies mention leadership as key to implementing the HPS framework (Rowling and Samdal, 2011; Langford *et al*., 2015). However, it is often not clear what constitutes an effective leadership role (McMullen *et al*., 2020; McNeish and Tran, 2020) and how it may differ between different school communities. HSCs engaged in the FLASH intervention indicated that they needed the time and support to determine what kind of leadership role was required to drive the capacity-building process. Within the limited research on leadership roles, adaptive leadership is mentioned as beneficial for implementing community-based approaches (Bertram *et al*., 2015). Such leadership builds on the notion that leaders assume a flexible role in identifying problems and opportunities, and in seeking shared values and agreement among different community stakeholders. During the FLASH intervention, HSCs demonstrated such leadership by focusing on bringing people together, finding shared values and opportunities appropriate to the context of a specific school and fostering collaborations. At the same time, HSCs sometimes indicated a lack of a sense of authority to act on their changing role, for example in asking people to actively join the HPS team. This highlights the need to invest in the commitment of formal school leaders for the successful implementation of the HPS framework (Betschart *et al*., 2022).

A notable challenge for HSCs and teachers throughout the FLASH intervention was involving parents and pupils. Research often cites participation as an important factor for implementation; however, it is well documented that eliciting participation in real life is ambitious since pupils and parents often lack motivation to participate on the topic of health and since projects often lack sufficient resources to set up large-scale participation (Deschesnes *et al*., 2003; Clelland *et al*., 2013; Bolton *et al*., 2016). Stakeholders in this study mentioned positive experiences with specific participatory tools, such as photovoice, and therefore deemed them helpful in encouraging participation. Previous studies that used photovoice also highlight appreciation for the flexible and creative nature of this tool (Warne *et al*., 2013; Trout *et al*., 2019). At the same time, HSCs and teachers experienced that, even with increased attention on fostering collaborations, the involvement of pupils and parents remained low. This might not be surprising as even stakeholders in projects with a single focus on participation note a similar struggle in eliciting large-scale participation (Lems *et al*., 2019; Anselma *et al*., 2020). Research on the participation and involvement of parents also suggests that schools struggle to engage families in general, not just on the topic of health (DeSpain *et al*., 2018; Kelty and Wakabayashi, 2020). A possible solution to achieving a stronger pupil and parent participation is by focusing on how participatory tools can best be ‘added-in’ to the curriculum and school structure instead of ‘added-on’ as additional (separate) activities (Driessen-Willems *et al*., 2022). For example, photovoice could become a recurring project in the curriculum and design thinking sessions as part of parent–teacher conferences. Moreover, by approaching the creation of positive attention for the Healthy School with an ‘add-in’ approach (e.g. through regular communication updates via familiar information channels), a wider audience of pupils and parents can be reached.

Finally, we observed that school personnel had the tendency to focus on separate, and sometimes disconnected, activities that are relatively easy to organize and can offer them ‘quick wins’, such as organizing a curriculum-based project or implementing an education program. Similar to other studies, experiences of stakeholders in our study suggest that this can be beneficial because starting small with activities that show quick results motivates them to seek opportunities for more complex organizational changes (Bartelink *et al*., 2019; Verdonschot, 2020). However, we also observed that stakeholders struggled to combine these separate activities into a comprehensive action plan that builds on the principles of an integral approach (WHO, 2009). This was evident from two experiences in particular, namely, (i) the struggle to go beyond stating behavioral rules for pupils when developing Healthy School policies and (ii) the lack of awareness about the importance of reflecting on the process of building community capacity. Deschesnes *et al*. (2003) also note that practitioners working in the (HPS) field have the tendency to ‘cherry-pick’ activities that meet the needs of the school. Although this enables flexibility and adaptability, being too inclusive risks leaving the impression that every health promotion activity fits within the HPS framework even if that activity is not ‘comprehensive’ in nature (i.e. simultaneously working on education, policy, environment etc.). To avoid this compartmentalization, Deschesnes *et al*. (2003) argue that it is important to make someone responsible for determining whether actions and activities in an action plan are comprehensive and complementary to each other and whether they build on the community’s needs and wants. Furthermore, they highlight that such an action plan is not a single-moment decision but a plan that can be adjusted based on the work completed and the reflections on the process. HSCs had this responsibility during FLASH, but as previously discussed, this was a new role for them.

### Implications for practice

Based on the facilitating and hindering factors identified in this study, two main implications for practice emerge. First, HSCs need professional development to become strong leaders. The importance of training leaders in (school) communities is well documented, but more research is needed on what strong leadership entails (Whelan *et al*., 2018; McNeish and Tran, 2020; Levin *et al*., 2021). This study highlights that an HSC needs to lead the coordination of the overall process of creating a Healthy School. Thus, an HSC who works toward building community capacity has the responsibility to serve as a central and visible point of contact while simultaneously ensuring that the process of creating a Healthy School is still moving ahead. Moreover, in order to facilitate the process of capacity building, a leader should also be able to identify and motivate other stakeholders for more specific leadership roles. Additionally, it is important to encourage the commitment of formal school leaders who can enable the HSC to take on such a role.

Second, we recommend that professionals outside the school provide continued support in the process of capacity building. Although the school itself should lead the process, the results of this study suggest that stakeholders appreciate an outside perspective to keep them on track in the capacity-building process. If an HSC has someone who proactively checks in with them on their progress and offers advice and support in a context-oriented manner, they potentially become better equipped to deal with barriers that may hinder the capacity-building processes, such as finding time for Healthy School responsibilities. In the Netherlands, there exists a system of Healthy School advisors, employed by regional public health services (Boot *et al*., 2010). However, little is known about how these advisors can support HSCs in an adaptive and context-oriented manner (Boot and De Vries, 2012). Moreover, results of this study suggest that HSCs might benefit from professional support in areas more specifically related to the capacity-building process, such as coaching on leadership and instigating organizational change. This type of support might also benefit schools if other issues emerge that capture stakeholder engagement, such as other health issues or other societal issues.

### Strengths and limitations

A strength of this study was the focus on the experiences and needs of stakeholders in real life, which enabled us to identify implications for practice. While this study was conducted within the Dutch context, results could also be of use to other countries because implementation issues and solutions raised by participants also apply to the HPS framework advocated by the WHO (Deschesnes *et al*., 2003).

Because of the explorative nature of the FLASH intervention in building community capacity in Dutch secondary schools, only four schools participated in it. We did include schools with varying characteristics, and while nuanced differences were observed between schools, participants across schools experienced similar barriers and facilitators in the capacity-building process. However, future research should investigate whether schools operating under different contexts experience different barriers and facilitators.

Finally, it is important to note that only a limited number of stakeholders were interviewed in each school, and the study lacked the pupil and parental perspective in one school. Therefore, the results might not represent the experiences and perceptions of the entire school community. This is especially the case in the school that was not able to involve pupils and parents in this study due to various adverse circumstances. However, we purposely included a mix of stakeholders per school, each with an active role in the capacity-building process in order to gain different perspectives. We saw that the perspectives between stakeholders were complementary to each other instead of contrasting, indicating that the different stakeholders together provided a fair representation of the capacity-building processes in their school.

## CONCLUSION

This study has demonstrated the importance of the strong leadership of HSCs in building community capacity. Strong leadership entails a more adaptive and dynamic leadership style where a coordinator keeps fostering collaborations, identifying resources and recognizing opportunities for others to take up leadership roles and participate. Because this was a new role for coordinators, they needed time and support to grow into it. Community members appreciated the tools used to facilitate participation, which helped them feel more involved. However, encouraging participation among a larger group of pupils and parents and finding ways to involve them throughout the different phases of the capacity-building process remained difficult. Gaining insight into the needs and wants of community members and the strengths of the school worked well for developing context-specific but separate activities. Coordinators can benefit from professional development to align jointly designed activities into a comprehensive action plan that can be embedded into Healthy School policies.

## Supplementary Material

daad115_suppl_Supplementary_Appendix_S1

daad115_suppl_Supplementary_Appendix_S2

## Data Availability

The raw data supporting the conclusions of this article will be made available by the authors upon reasonable request, without undue reservation.

## Notes

Trial registration: ISRCTN67201841; date registered: 9 May 2019, retrospectively registered.
