# Supplementary material for: Increasing community capacity to improve the implementation of Health Promoting Schools: barriers and facilitators from the FLASH intervention
Source: Health Promot Int. 2023 Sep 30;38(5):daad115. doi: 10.1093/heapro/daad115 (PMC10541852; doi:10.1093/heapro/daad115)
Supplement: daad115_suppl_Supplementary_Appendix_S1 [file daad115_suppl_supplementary_appendix_s1.docx]

**Appendix 1 – Overview of the adoption and implementation of capacity-building strategies per school**

This table is based on the reported outcomes of the impact evaluation in the publication ‘van Dongen B.M., de Vries I.M., Ridder M.A.M., de Boer M., Steenhuis I.H.M., Renders C.M. Building community capacity to stimulate physical activity and dietary behavior in Dutch secondary schools: Evaluation of the *FLASH* intervention using the REAIM framework. *Frontiers in Public Health*. *2022.* 10:926465’

|  | **School 1** | **School 2** | **School 3** | **School 4** |
| --- | --- | --- | --- | --- |
| **Adoption – willingness to/from:** | | | | |
| ***Strategy 1: Identifying leadership*** | | | | |
| *Facilitate HSC* | One HSC was facilitated continuously for the first two years of the intervention. A change occurred in Year 3. The new HSC was engaged in the intervention for ¾ of Year 3. | One HSC was facilitated continuously for the duration of intervention. | One HSC was facilitated continuously in Year 1. A change of HSC occurred in Year 2. The new HSC was facilitated continuously in Years 2 and 3. | One HSC was facilitated continuously for the duration of intervention. |
| ***Strategy 2: Creating a participatory school culture*** | | | | |
| *Use participatory methods* | Design thinking: Yes, start of Year 3  *Photovoice*: Yes, in Years 1 and 2 | Design thinking: Yes, end of Year 2  *Photovoice*: Yes, in Years 1 and 2 | Design thinking: Yes, end of Year 2  *Photovoice*: Yes, in Years 1 and 2 | Design thinking: Yes, start of Year 3  *Photovoice*: Yes, in Years 1 and 2 |
| ***Strategy 3: Designing tailored activities*** | | | | |
| *Develop action plan for implementation budget* | Yes, but the first action plan was rejected based on limited input from DT session and lack of integral approach. Adjustments were made. | Yes, together with colleague, and based on input from DT session | Yes, together with colleague, and based on input from DT session | Yes, together with colleague, and based on input from DT session |
| *Facilitate changes for the Healthy School approach* | Yes, by a manager and teacher. Concerns were expressed with regard to willingness of canteen staff. | Yes, but priorities shifted due to increasing pupil numbers | Yes, but with the note that certain changes require approval of all three schools in the building. | Yes, but resources limited due to small size of the school |
| ***Strategy 4: Creating local networks*** | | | | |
| *From local organizations* | Expert from educational organization: Role was facilitated throughout Years 1 and 2 and for 1/3 of Year 3. One person held this position continuously.  Expert from municipal health service: One person was facilitated from October through March of Year 1. A new person was facilitated from May in Year 1 until January in Year 3. In all, the experts organized 15 coaching sessions for the HSCs, in collaboration with the principal researchers. | | | |
| **Implementation – extent to which:** | | | | |
| ***Strategy 1: Identifying leadership*** | | | | |
| *HSC hours were used* | HSC indicated often not using allocated weekly hours, due to other priorities | HSC indicated using allocated weekly hours half of the time | HSC indicated using allocated weekly hours half of the time | HSC indicated using allocated weekly hours most of the time |
| *Other leaders were motivated* | - First HSC also had managerial role, no full support from other managers. School director not motivated  - Workgroup formed with biology and economics teacher  - PR employee intrinsically motivated to improve visibility of Healthy School  - Second HSC (no managerial role) got support from one manager as sparring partner. As a teacher this HSC used their own pupils for leadership | - School leader: motivated, better balance between responsibilities  - PE colleague as sparring partner  - Biology teacher: connection to climate change workgroup and to pupil council  - Motivated canteen employee for healthy food environment  - Pupil and parent council difficult to reach for Healthy School | - Little support from management due to new school structure and formal organization in Year 1. Second HSC more assertive in instigating dialogue  - Biology and two health/well-being teachers formed workgroup  - PR employee intrinsically motivated to improve visibility of Healthy School  - No pupil or parent council were in place, pupils/parents somewhat resistant towards the school | - School leader: active agenda setting  - PE and biology teacher executing ideas  - Custodian: spokesperson between parties (pupils, teachers, managers)  - Pupil and parent council: health as regular topic on their agendas |
| *Coaching sessions were attended* | 12 out of 15 (average experience score of 7.6) | 15 out of 15 (average experience score of 6.9) | 13 out of 15 (average experience score of 7.5) | 13 out of 15 (average experience score of 8.3) |
| ***Strategy 2: Creating a participatory school culture*** | | | | |
| *Community participated in design thinking sessions* | 22 participants in total: 3 teachers, 1 team leader, 1 PR employee, 2 parents, 7 pupils, the HSC, and 1 local expert | 13 participants in total: 3 teachers, 1 team leader, 2 parents, 2 pupils, the HSC, and 1 local expert | 5 participants in total: 1 team leader, 2 parents, 1 pupil, the HSC, and 1 local expert | 17 participants in total: 3 teachers, 1 school manager, 2 parents, 7 pupils, the HSC, and 1 local expert |
| *pupils participated in Photovoice* | 7 pupils engaged in 3 1-hour sessions | 1 second-year class engaged in 4 2-hour sessions | 2 second-year classes engaged in 1 afternoon session | 2 second-year classes engaged in 4 2-hour sessions |
| ***Strategy 3: Designing tailored activities*** | | | | |
| *Action plan was carried out* | Part of the plan was carried out, aimed largely at incidental activities instead of structural changes. | Plan was carried out, but the impact remained limited due to implementation issues concerning reach. | Plan was largely carried out, with revisions necessitated by contextual factors. | Plan was not carried out during the intervention, but an adjusted activity was conducted. |
| *Additional Healthy School activities were set up* | - A water tap was installed. - Changes were attempted in the school canteen and in-house retail shop run by pupils - The school yard was re-designed with school resources. | - Changes were made to the nutrition policy. - Successful existing activities were adjusted and continued. - Willingness to facilitate activities in the new pre-vocational school location was limited. | - A water tap was installed.  - Health education and a Healthy School canteen were implemented out in the new location. | - A second activity created in the DT session was implemented with school resources. - Changes were made to the school canteen and screen-time policies aimed at reducing sedentary behavior were adopted. |
| ***Strategy 4: Creating local networks*** | | | | |
| *Connections were established with local organizations* | - Collaboration with organizations for internships, but no connections established with regard to health promotion - School management in contact with local supermarket about waste reduction, but not about health promotion | - Structural contact with sports organizations by PE teacher, who also uses available sports equipment from the Landstede Group as a resource - Conversation initiated with municipality about physical environment, but with limited impact | Collaboration initiated between the school and neighborhood sports coaches, but additional support needed to make the collaboration more profitable for both parties | - Structural contact with sports organizations by the PE teacher - Structural collaboration with local municipal youth team |
