# Supplementary material for: Increasing community capacity to improve the implementation of Health Promoting Schools: barriers and facilitators from the FLASH intervention
Source: Health Promot Int. 2023 Sep 30;38(5):daad115. doi: 10.1093/heapro/daad115 (PMC10541852; doi:10.1093/heapro/daad115)
Supplement: daad115_suppl_Supplementary_Appendix_S2 [file daad115_suppl_supplementary_appendix_s2.docx]

**Appendix 2 – Example questions for adult participants and pupils per capacity-building strategy**

| *Capacity-building strategy* | *Example questions for adult participants* | *Example questions for pupils* |
| --- | --- | --- |
| 1: Leadership | Who are the current leaders and what are their roles? To what extent are the appointed leaders, key figures and influential members in the school willing to commit to create a Healthy School? Have you experienced any changes? | Who takes charge when it comes to the Healthy School (staff, pupils and parents)? What do they do? Should anyone else take charge? |
| 2:Participatory school culture | What is your ideal Healthy School? What is the general attitude of the community towards healthy physical activity and dietary behavior in the school? To what extent are various stakeholders willing to participate in developing and implementing activities? Have you experienced any changes? | What is your ideal Healthy School? Is health an important topic in your school and why? Does the school ask pupils/ parents/ staff what it should do and how? |
| 3: Tailored activities | What is currently being done in the Healthy School and what do you think about this? To what extent are other people in the school familiar with these activities? To what extent do people know about the prevalence of this health problem and is this information used to prioritise and tailor activities? Have you experienced any changes? | What is happening in the school to promote healthy physical activity and dietary behavior? Does everybody know this is happening? What do you think about these activities? |
| 4: Local networks | To what extent are local resources, collaborations and opportunities available to continue supporting efforts around the theme? Have you experienced any changes? | Who outside the school helps the school to create your ideal Healthy School? |
